# Functional Dichotomy between NKG2D and CD28-Mediated Co-Stimulation in Human CD8^+^ T Cells

**DOI:** 10.1371/journal.pone.0012635

**Published:** 2010-09-09

**Authors:** Kamalakannan Rajasekaran, Va Xiong, Lee Fong, Jack Gorski, Subramaniam Malarkannan

**Affiliations:** 1 Laboratory of Molecular Immunology, Blood Research Institute, Milwaukee, Wisconsin, United States of America; 2 Laboratory of Molecular Genetics, Blood Research Institute, Milwaukee, Wisconsin, United States of America; 3 Department of Medicine, Medical College of Wisconsin, Milwaukee, Wisconsin, United States of America; New York University, United States of America

## Abstract

Both CD28 and NKG2D can function as co-stimulatory receptors in human CD8^+^ T cells. However, their independent functional contributions in distinct CD8^+^ T cell subsets are not well understood. In this study, CD8^+^ T cells in human peripheral blood- and lung-derived lymphocytes were analyzed for CD28 and NKG2D expression and function. We found a higher level of CD28 expression in PBMC-derived naïve (CD45RA^+^CD27^+^) and memory (CD45RA^−^CD27^+^) CD8^+^ T cells (CD28^Hi^), while its expression was significantly lower in effector (CD45RA^+^CD27^−^) CD8^+^ T cells (CD28^Lo^). Irrespective of the differences in the CD28 levels, NKG2D expression was comparable in all three CD8^+^ T cell subsets. CD28 and NKG2D expressions followed similar patterns in human lung-resident GILGFVFTL/HLA-A2-pentamer positive CD8^+^ T cells. Co-stimulation of CD28^Lo^ effector T cells via NKG2D significantly increased IFN-γ and TNF-α levels. On the contrary, irrespective of its comparable levels, NKG2D-mediated co-stimulation failed to augment IFN-γ and TNF-α production in CD28^Hi^ naïve/memory T cells. Additionally, CD28-mediated co-stimulation was obligatory for IL-2 generation and thereby its production was limited only to the CD28^Hi^ naïve/memory subsets. MICA, a ligand for NKG2D was abundantly expressed in the tracheal epithelial cells, validating the use of NKG2D as the major co-stimulatory receptor by tissue-resident CD8^+^ effector T cells. Based on these findings, we conclude that NKG2D may provide an expanded level of co-stimulation to tissue-residing effector CD8^+^ T cells. Thus, incorporation of co-stimulation via NKG2D in addition to CD28 is essential to activate tumor or tissue-infiltrating effector CD8^+^ T cells. However, boosting a recall immune response via memory CD8^+^ T cells or vaccination to stimulate naïve CD8^+^ T cells would require CD28-mediated co-stimulation.

## Introduction

CD28 and NKG2D can function as co-stimulatory receptors in CD8^+^ T cells [1]. However, their functional contribution in T cell activation is not well understood. NKG2D is an important activating receptor of NK cells [2] and has been defined as a co-stimulatory molecule for TCR- αβ and TCR-γδ CD8^+^ T cells [1]. In humans, the ligands for NKG2D are major histocompatibility class I-related chain A/B (MICA/B) and UL-16 binding proteins 1–4 (ULBP1-4). ULBP4 is also known by the other name lymphocyte effector cell toxicity-activating ligand, LETAL [3]. While ULBP 1,2 and 3 are GPI-linked proteins, the more divergent LETAL is a transmembrane protein [4], [5]. Ligand expression is restricted or absent on normal tissues but is induced in response to various stresses and in some pathological conditions including epithelial-derived tumors [6]. In human T cells, NKG2D exclusively associates with DAP10 and not DAP12 and its co-stimulation enhances proliferation, survival, expression of activation markers and production of IFN-γ, TNF-α and IL-2 [7], [8]. However, NKG2D-mediated activation of CD8^+^ T cell effector functions in the absence of CD3-mediated stimulation is controversial [8], [9]. Earlier studies suggest that activated cytotoxic T lymphocytes (CTLs), intra-epithelial lymphocytes (IELs) and γδ T cells could trigger cytotoxicity through NKG2D stimulation alone, although this was insufficient to generate cytokine production [10]. Other studies show that NKG2D does not co-stimulate proliferation, cytotoxicity or cytokine production in naïve or activated human CTL [11]. These contradictory findings indicate that the role of NKG2D in regulating CD8^+^ T cell effector functions is not fully understood.

CD28 recognizes the B7 family of ligands (B7.1-CD80, B7.2-CD86, ICOS-L, PD-L1, PD-L2, B7-H3 and B7-H4). Interaction of CD28 with CD80 and CD86 are critical for naïve CD8^+^ T cell activation [12]. CD28 recruits PI3K-p85α through its cytoplasmic YMNM motif and synergizes with TCR/CD3-mediated signaling cascades [13]. Earlier studies have shown that a co-stimulation via CD28 is essential for IL-2 production and IL-2Rα (CD25) expression [14]. CD28 is also known to regulate the threshold of TCR-mediated activation and significantly reduce the number of TCR-epitope/MHC interactions needed to promote the proliferation, clonal expansion, differentiation and survival of T cells [15]. CD28 co-stimulation can also augment the expression of Bcl-x_L_, a key cell survival molecule that prevents the induction of apoptosis [16]. CD8^+^ T cells can be divided into CD28 low (CD28^Lo^) and CD28 high (CD28^Hi^) subsets. In particular, using phenotypic markers, the CD28^Lo^ subset has been determined to be effector, while the CD28^Hi^ subset as naïve/memory CD8^+^ T cells [8]. A dichotomy in the CD28 expression and a functional requirement for any other co-stimulatory receptors such as NKG2D for CD28^Lo^ effector T cells is not well understood.

In this study, we found that the expression of NKG2D was not altered among CD8^+^ T cell subsets. However, NKG2D but not CD28 functioned as an essential co-stimulatory receptor in human peripheral blood mononuclear cell (PBMC)-derived CD28^Lo^CD45RA^+^CD27^−^ CD8^+^ effector T cell population. CD28 expression was high in naïve/memory CD8^+^ T cells, where it worked as the primary co-stimulatory molecule. Differences in the expression pattern of CD28 were also observed in the lung-resident influenza-specific CD8^+^ T cell subsets. Uniquely, effector CD8^+^ T cells generated a higher level of IFN-γ and TNF-α in response to NKG2D-mediated co-stimulation. Conversely, irrespective of its normal expression, NKG2D did not co-stimulate the generation of IFN-γ, TNF-α or IL-2 in CD28^Hi^ memory CD8^+^ T cells. For IL-2 generation, CD28-mediated co-stimulation was obligatory and its production was limited only to the CD28^Hi^ naïve/memory subsets. MICA was abundantly expressed within the tracheal epithelial cells. These results provide critical insights into the functional dichotomy of co-stimulatory receptors in distinct CD8^+^ T cells.

## Results

### Differential expression of CD28 and NKG2D in human CD8^+^ T cells

**Figure 1:**
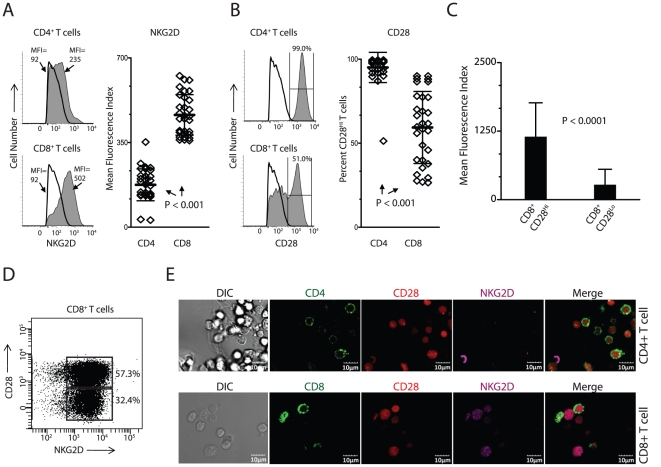
Expression of NKG2D and CD28 on human CD3^+^CD4^+^ and CD3^+^CD8^+^ T cells. **A**) Analysis of NKG2D expression on CD3**^+^**CD4**^+^** and CD3**^+^**CD8**^+^** T cells by flow cytometry. Human PBMC were stained for CD3, CD4 or CD8 along with NKG2D or CD28. Background isotype control (open histograms) and NKG2D (grey histograms) stainings are shown. The mean fluorescence index (MFI) for NKG2D (N = 30) among the CD3**^+^**CD4**^+^** and CD3**^+^**CD8**^+^** T cells were compared and presented in the right panel. **B**) Similar analysis was performed for the expression of CD28 on CD3**^+^**CD4**^+^** and CD3**^+^**CD8**^+^** T cells. Open histograms represent negative controls. Since the expression of CD28 on CD8^+^ T cells was biphasic, a comparison of the percentage of CD28^Hi^CD4^+^ and CD28^Hi^CD8^+^ T cells was performed (N = 30). **C**) CD28 expression among CD3**^+^**CD8**^+^** T cells falls into two distinct subsets. Differences between the MFI of CD28**^Hi^** and CD28**^Lo^**CD8**^+^** T cells are shown. **D**) Co-expression of CD28 and NKG2D in CD3**^+^**CD8**^+^** T cells. **E**) Confocal microscopy analyses of the expression of CD28 and NKG2D on CD4**^+^** and CD8**^+^** T cells. PBMC were either stained for CD4 or CD8. They were also stained for CD28 and NKG2D. The stained cells were visualized by confocal microscopy and expression of each receptor was visualized individually and merged. Arrow heads indicate limited positivity of NKG2D in CD4^+^ T cells. P values for A–C were calculated using Student's t-test.

CD28 and NKG2D have co-stimulatory functions in T cells (9–12). Both CD28 and NKG2D/DAP10 recruit PI3K to propagate their signals. However, a functional necessity for T cells to express two similar co-stimulatory receptors is not well understood. Moreover, qualitative differences between NKG2D and CD28-mediated co-stimulation in cytokine production are yet to be defined. To understand the differential abilities of these two co-stimulatory receptors, we first analyzed their expression patterns. Human PBMC-derived CD3^+^CD8^+^ and CD3^+^CD4^+^ T cells (N = 30) revealed that the majority of the CD8^+^ T cells expressed higher levels of NKG2D and its expression in CD4^+^ T cells was low (**Figure 1A**). The difference in the expression of NKG2D between these two T cell subsets was statistically significant (**Figure 1A**; P<0.001). The majority of the PBMC-derived CD3^+^CD4^+^ T cells uniformly expressed CD28 at a high level (**Figure 1B**; N = 30). However, CD28 expression in CD8^+^ T cells followed a biphasic pattern with the CD28 high population (CD28^Hi^) ranging from 25 to 80% (**Figure 1B**; P<0.001). Remaining CD8^+^ T cells expressed lower levels of CD28 (CD28^Lo^). The difference in the mean fluorescence index (MFI) of CD28 between the CD28^Lo^ and CD28^Hi^ T cells was significant (**Figure 1C**; P<0.0001). In contrast, expression of NKG2D did not vary between the CD28^Hi^ and CD28^Lo^ CD8^+^ T subsets (**Figure 1D**). Expression of CD28 and NKG2D in PBMC-derived CD4^+^ and CD8^+^ T cells were further confirmed by confocal microscopy (**Figure 1E**). Our results indicate both CD4 and CD8 T cells expressed CD28. However, the constitutive expression of NKG2D could be observed only in the CD8^+^ T cells.

### The functional status of CD8^+^ T cells is determined by CD28 but not NKG2D expression

**Figure 2:**
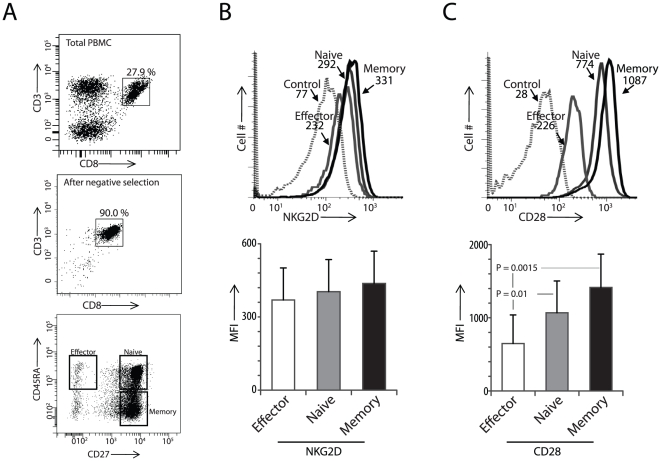
Expression of CD28 but not NKG2D is low in the PBMC-derived CD45RA^**+**^CD27^**−**^ effector CD8^**+**^ T cells. **A**) CD3**^+^**CD8**^+^** T cells were classified into naïve, effector and memory subsets based on their CD27 and CD45RA expression. **B**) Flow cytometry analysis of the expression of NKG2D and **C**) CD28 in the effector, naïve and memory CD8**^+^** T cells. Histograms in B and C (open-effector, grey-naïve and black-memory) depict the expression of NKG2D and CD28 in the different functional subsets of CD8**^+^** T cells. Dashed line histograms represent negative controls. Nine independent samples were used to obtain data presented in B and C. P values were calculated using Student's t-test.

To further understand the expression patterns of NKG2D and CD28, PBMC-derived CD8^+^ T cells were separated based on their functional status using CD45RA and CD27 receptor expression. CD8^+^ T cells were negatively selected, stained and phenotypically separated into naïve (CD45RA^+^CD27^+^), effector (CD45RA^+^CD27^−^) and memory (CD45RA^−^CD27^+^) T cells (**Figure 2A**). These subsets were analyzed for NKG2D and CD28 expression. Our results indicate that there were no differences in the expression of NKG2D between the naïve, effector and memory CD8^+^ T cells (**Figure 2B**). However, the expression of CD28 was found to be significantly high on the memory CD8^+^ T cells. Naïve CD8^+^ T cells expressed moderate levels of CD28. The effector CD8^+^ T cells expressed the least amount of CD28 when compared to the memory (P = 0.0015) or naïve (P = 0.01) CD8^+^ T cells (**Figure 2C**). These differences were statistically significant and indicate a possible functional role for NKG2D in CD8^+^ effector T cells.

### Human lung-resident, antigen-specific effector CD8^+^ T cells express lower levels of CD28

**Figure 3:**
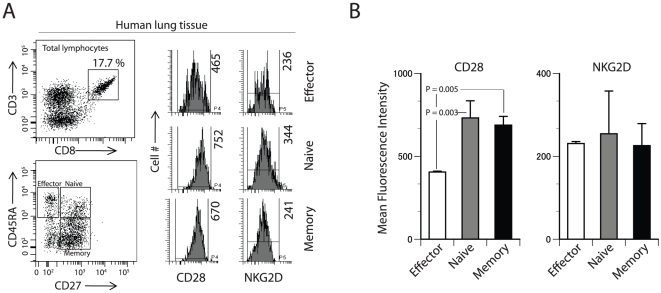
Human lung-derived CD45RA^**+**^CD27^**−**^ effector CD8^**+**^ T cells express low levels of CD28. **A**) CD3**^+^**CD8**^+^** T cells were classified into naïve, effector and memory cells based on CD27 and CD45RA surface expression. These subsets were analyzed for the expression levels (MFI) of CD28 and NKG2D. **B**) Comparison of CD28 (left histogram) and NKG2D (right histogram) in effector (open bar), naïve (grey bar) and memory (black bar) CD8**^+^** T cells. Four independent samples were analyzed to obtain data presented in B and the P values were calculated using Student's t-test.

Since lymphocytes derived from the PBMC may not represent the functional status of T cells in the tissue environment, we extended our analyses to human lung-derived CD8^+^ T cells. We obtained lung tissues from cadaveric organ donors. Lymphocytes were isolated and the expression levels of NKG2D and CD28 were analyzed on CD8^+^ T cells. Phenotypic separation based on CD45RA and CD27 helped to separate the naïve, effector and memory CD8^+^ T cells (**Figure 3A**). Analyses in multiple individuals (N = 4) demonstrate that the lung-derived CD45RA^+^CD27^−^effector CD8^+^ T cells expressed a lower level of CD28 while its level was significantly higher in CD45RA^+^CD27^+^ naïve (P = 0.003) and CD45RA^−^CD27^+^ memory (P = 0.005) CD8^+^ T cells (**Figure 3B**). However, no such differences were seen in the levels of NKG2D expression among these subsets. Based on these results, we conclude that NKG2D may have an expanded co-stimulatory role to play along with CD28 in CD45RA^+^CD27^−^ effector CD8^+^ T cells.

**Figure 4:**
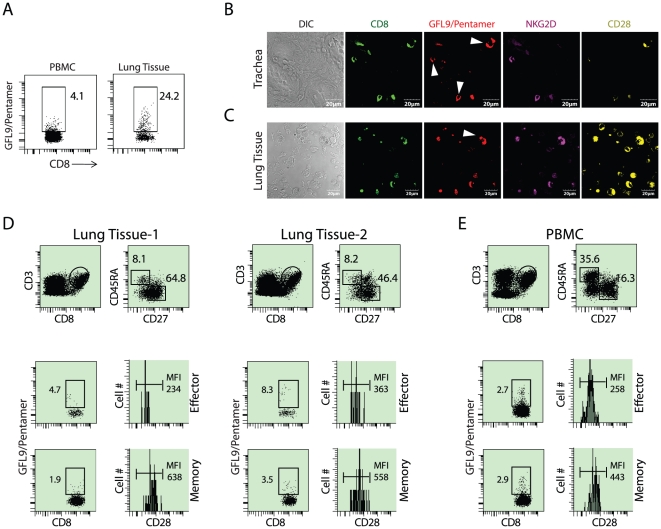
Analysis of CD28 expression on influenza-specific (M1 peptide, GFL9) CD8^**+**^ T cells in PBMC and lung. **A**) Human PBMC and lung-derived lymphocytes were stained with anti-CD3, anti-CD8 and GFL9/HLA-A2 pentamer to detect antigen-specific CD8**^+^** T cells. Gated populations indicate percent GFL9 epitope-specific CD8**^+^** T cells. CD28 expression is higher in antigen-specific memory CD8^+^ T cells. **B)** GFL9/HLA-A2 pentamer-positive tracheal and **C)** lung tissue-resident CD8**^+^** T cells were analyzed for CD28 and NKG2D expression using confocal microscopy. Arrow heads mark some of the pentamer-positive CD8^+^ T cells. **D)** Lung tissue and **E)** PBMC were stained for CD27 and CD45RA in order to classify them into effector and memory cells. These cells were stained with anti-CD28 and GFL9/HLA-A2 pentamer. Histograms show the level of CD28 expression among the pentamer-positive effector or memory CD8^+^ subsets. Numbers in the histograms represent the MFI of CD28 expression.

To investigate whether antigen-specific CD8^+^ T cells also show similar CD28 expression patterns, we stained the lung-derived lymphocytes with peptide-loaded MHC Class I pentamer (**Figure 4**). HLA-A2 positive individuals generate a strong CD8^+^ memory T cell response to an immunodominant epitope, (GILGFVFTL; GFL9), from influenza virus M1 protein [17]. The frequency of GFL9-specific CD8^+^ T cells is high enough to allow direct visualization using GFL9/HLA-A2 pentamers [18]. Therefore, we used flow cytometry to analyze antigen-specific CD8^+^ T cells from PBMC and lung-derived lymphocytes using GFL9/HLA-A2 pentamer (**Figure 4A**). This analysis was extended to cryosections derived from human trachea and lung tissue (**Figure 4B and 4C**). Abundant GFL9/HLA-A2 pentamer-positive CD8^+^ T cells could be detected in these cryosections using confocal microscopy. Staining of tracheal sections consistently indicated that GFL9/HLA-A2 pentamer-positive T cells preferentially expressed NKG2D. However, the number of CD28 expressing CD8+ cells was lower compared to that of NKG2D expressing CD8+ T cells (**Figure 4B**). However, pentamer-positive T cells in the lung tissue expressed both CD28 and NKG2D (**Figure 4C**). Thus, it is possible that trachea contains more effector CD8^+^ T cells while the memory cells home in the lung tissue. To further determine the subset specific expression of CD28 among pentamer-positive cells, lung-derived CD3^+^CD8^+^ T cells were analyzed for CD45RA, CD27 and CD28 receptors (**Figure 4D**). While the PBMC-derived lymphocytic populations contained a considerable number of CD45RA^+^CD27^−^ effector T cells (**Figure 4E**), lung tissues possessed only a few of this subset and a higher number of CD45RA^−^CD27^+^ memory T cells (**Figure 4D**). This higher frequency of CD28^Hi^ memory CD8^+^ T cells in the lung tissue strongly correlated with our confocal data (**Figure 4C**). Together, our results show influenza-derived GFL9/HLA-A2-restricted CD45RA^+^CD27^−^ effector T cells express lower levels of CD28 compared to that of CD45RA^−^CD27^+^ memory T cells. Based on these results, we conclude antigen-specific, tissue-resident CD45RA^+^CD27^−^ CD8^+^ T cells express lower levels of CD28 indicating an expanded co-stimulatory role for NKG2D.

### MICA is expressed in tracheal epithelial cells

**Figure 5:**
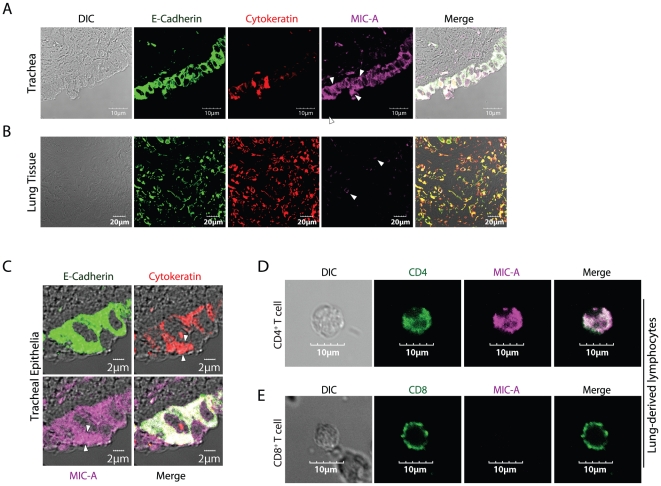
MICA is expressed in human tracheal epithelial cells. Paraffin sections of human **A**) trachea and **B**) lung tissue were processed and stained for MICA and the epithelial cell markers E-Cadherin and cytokeratin. **C**) Enlarged view of tracheal sections showing the intracellular localization of MICA (Arrow heads). **D**) CD4^+^ but not **E**) CD8 T cells express MICA. Lung-derived lymphocytes were stained for CD4**^+^** and CD8**^+^** T cells. CD4**^+^** and CD8**^+^** T cells were further stained and analyzed for MICA expression through confocal microscopy. Data shown are one representative image of seven independent sections analyzed for each lung tissue and trachea.

Successful co-stimulation via NKG2D in lung-derived CD45RA^+^CD27^−^ effector CD8^+^ T cells requires the expression of ligands at these sites. Human NKG2D recognizes stress-inducible MICA, MICB and ULBPs [1], [19]. Tracheal and lung sections from human cadavers were stained with anti-MICA antibody. Antibodies to E-Cadherin and cytokeratin uniquely marked the stratified columnar epithelial cells in the trachea (**Figure 5A**) and these cells showed a strong expression of MICA. Staining of lung tissues also indicated the presence of MICA positive cells; however, compared to the tracheal epithelial cells this staining was only moderately intense (**Figure 5B**). MICA expression was found both in the cell surface and intracellularly; with considerable amount of the MICA restricted to intracellular compartments. MICA found within epithelial cells co-localized with cytokeratin (**Figure 5C**). In this context, it is important to note that the cadaveric donors were not known to have any respiratory illnesses prior to their death. Structures below the epithelial layer such as basement membrane, submucosal layer, areolar and the connective tissue or the cartilage did not express MICA. While the columnar epithelial cells showed a relatively uniform intracellular expression of MICA, there were places where its expression was concentrated in a polarized fashion (arrow heads). These polarized spots could be seen both on the apical and basal side of the epithelial cells (**Figure 5C**).

The number of cells that were MICA positive was fewer in the lung tissue compared to the trachea. These MICA positive cells were circular and appeared to be lymphoidal. Therefore, we extended our analyses to lymphocytes. Our results show lung-derived CD4^+^ T cells were strongly positive for MICA (**Figure 5D**), while the CD8^+^ T cells were not (**Figure 5E**). Based on these results, we conclude that MICA is present in the upper respiratory tract, albeit intracellularly. We predict a cellular stress such as influenza infection would initiate the transport of MICA from their cytoplasmic stores to the basement membrane of epithelial cells. Thus, CD28^Lo^ antigen-specific CD45RA^+^CD27^−^ effector CD8^+^ T cells can utilize NKG2D as a co-stimulatory receptor by recognizing MICA on infected tracheal epithelial cells along with antigenic epitopes presented on MHC class I molecules.

### NKG2D plays a critical expanded co-stimulatory role in CD28^Lo^ effector CD8^+^ T cells for cytokine production

**Figure 6:**
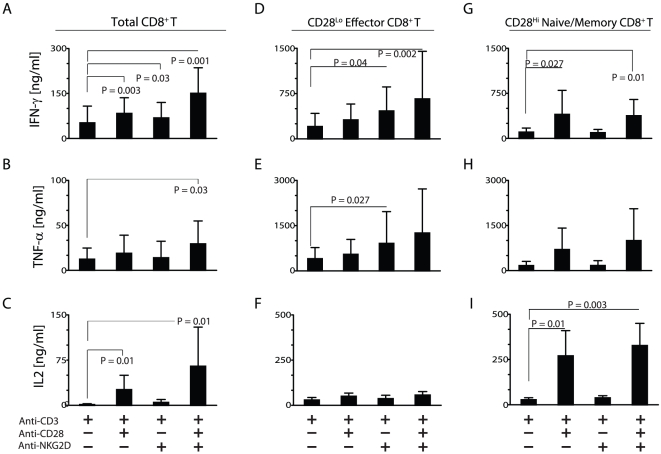
Efficiency of CD28 and NKG2D co-stimulation in cytokine/chemokine production. Negatively selected CD8**^+^** T cells (N = 10) or sorted CD28^Lo^ and CD28^Hi^ CD8**^+^** T cells (N = 5) were activated with plate-bound antibodies in different combinations. Quantities of IFN-γ, TNF-α and IL-2 were estimated in the culture supernatants using multiplex assays. Cytokine generation from total CD8^+^ (**A–C**), effector CD28^Lo^ (**D–F**) and naïve/memory CD28^Hi^ (**G–I**) CD8^+^ T cells are shown. Bar diagram represents the mean ± standard deviation of cytokine production. P values were calculated using paired t-test.

Presence of MICA in the tracheal epithelial cells and the reduced levels of CD28 in effector CD8^+^ T cells suggest an essential co-stimulatory role for NKG2D at the site of infection. To determine their independent functional contributions of CD28 and NKG2D, we sorted the total CD3^+^CD8^+^ T cells from human PBMC and stimulated them with plate-bound anti-CD3, anti-CD28 and anti-NKG2D mAb alone or in combinations. Culture supernatants were tested for the levels of IFN-γ, TNF-α or IL-2. Results presented in **Figure 6A–C** demonstrate anti-CD3 alone mediated some levels of IFN-γ and TNF-α. Anti-CD28 or anti-NKG2D mAb alone did not result in any cytokine production (data not shown). Co-stimulation via CD28 significantly increased the levels of IFN-γ and IL-2 (P = 0.003 and 0.01, respectively). Co-stimulation via NKG2D also increased the levels of IFN-γ production (P = 0.03). Combined stimulation via CD28 and NKG2D along with anti-CD3 significantly augmented the production of IFN-γ (P = 0.001) TNF-α (P = 0.03) and IL-2 (P = 0.01). This expanded effect of CD28 and NKG2D co-stimulation was evident only when the assay was performed on negatively selected CD3^+^CD8^+^ T cells but not total PBMC (data not shown). This could be attributed to the preferential expression of NKG2D on CD8^+^ over CD4^+^ T cells and the relative frequency of CD8^+^ T cells (10–15%) in the total PBMCs. Another possible explanation could be an increased expression of NKG2D on CD8^+^ T cells in response to CD3/CD28-mediated activation. However, our results indicated that NKG2D expression levels remained unchanged with or without stimulation (data not shown).

To determine the functional contributions of CD28 and NKG2D on CD8^+^ T cell subsets, we sorted CD8^+^ T cells into CD28^Lo^ effector and CD28^Hi^ memory and tested their ability to respond to anti-CD28 or anti-NKG2D-mediated co-stimulation in the presence of anti-CD3 mAb-mediated activation (**Figure 6D–I**). Cytokines in the culture supernatants were used as a measure of co-stimulation. CD28^Lo^ effector T cells were more efficient in generating IFN-γ (**Figure 6D**; P = 0.04) and TNF-α (**Figure 6E**; P = 0.027) when co-stimulated through NKG2D receptor. However, a co-stimulation through CD28 did not result in a significant increase in the generation of these cytokines when compared to anti-CD3 mAb-mediated activation alone. CD28^Lo^ effector T cells did not generate any IL-2 with anti-CD3 mAb activation (**Figure 6F**). Further, neither CD28 nor NKG2D-mediated co-stimulation could activate IL-2 production in CD28^Lo^ effector T cells (**Figure 6F**).

**Figure 7:**
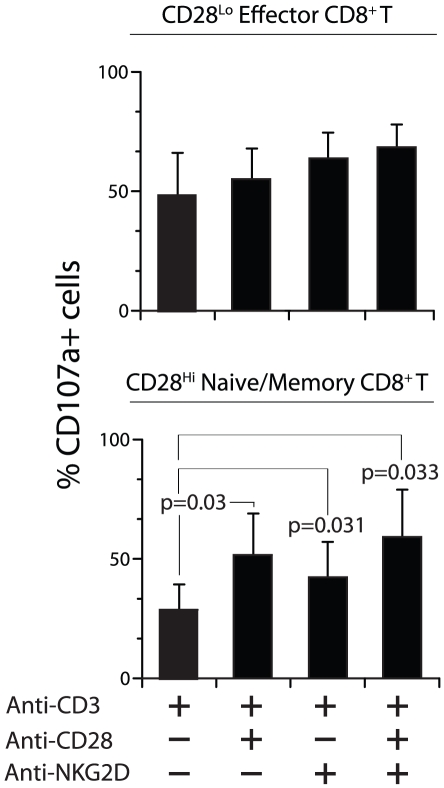
Influence of CD28 and NKG2D-mediated co-stimulation in cytotoxicity. Negatively selected CD3**^+^**CD8**^+^** T cells were further sorted based on their CD28 expression levels into CD28^Lo^ and CD28^Hi^ CD8**^+^** T cells. These cells were activated with plate-bound antibodies directed against CD3, CD28 and NKG2D in different combinations. Cell surface expression of CD107a was analyzed through flow cytometry and used as a measure of cytotoxicity. Three independent samples were used to obtain data and P values were calculated using paired t-test.

Interestingly, CD28^Hi^ memory T cells responded well for CD28-mediated co-stimulation in generating IFN-γ (**Figure 6G**; P = 0.027), TNF-α (**Figure 6H**) and IL-2 (**Figure 6I**; P = 0.01). In particular, CD28^Hi^ memory T cells generated higher levels of IL-2 in response to CD28-mediated co-stimulation. A difference in the ability of CD28 to preferentially co-stimulate CD28^Hi^ memory T cells could be logically attributed to the expression levels of CD28. However, irrespective of the comparable levels of NKG2D expression between CD28^Lo^ and CD28^Hi^ CD8^+^ T cells, the latter minimally responded to NKG2D-mediated co-stimulation (**Figure 6G–I**). The cytokine productions in the sorted CD28^Lo^ or CD28^Hi^ CD8^+^ T cells were consistently higher compared to that of unsorted CD8^+^ T cells. This difference could be due to a change in the relative frequency of CD28^Lo^CD8^+^ or CD28^Hi^CD8^+^ T cells after sorting. We also analyzed the requirement of CD28 or NKG2D-mediated co-simulations in the release of cytotoxic granules. Human PBMC-derived CD8^+^ T cells were activated with indicated combinations of mAbs and the granule release was quantified through CD107a expression. CD28^Hi^ memory CD8^+^ T cells were comparatively less effective than CD28^Lo^ effector T cells in expressing CD107a after CD3-mediated activation (**Figure 7**). However, CD107a expression did not differ between different combinations of mitogenic antibody stimulations in the CD28^Lo^ effector CD8^+^ T cells. In contrast, co-stimulation via NKG2D (P = 0.031), CD28 (P = 0.03) or both (P = 0.033) significantly increased the granule release in the CD28^Hi^ naïve/memory CD8^+^ T cells. Thus, we conclude both CD28 and NKG2D-mediated co-stimulations can significantly influence the cytotoxicity in CD28^Hi^ naïve/memory CD8^+^ T cells.

## Discussion

NKG2D and CD28 function as major co-stimulatory receptors in CD8^+^ T cells [9]. However, the differential expression of CD28 and the functional relevance of NKG2D in CD28^Lo^ CD8^+^ T subset are not well understood. Our results indicate that the expression of CD28 was high in PBMC-derived memory and naïve CD8^+^ T cells; while, its expression was significantly lower in effector CD8^+^ T cells. This pattern was also observed in human lung-derived CD8^+^ T cell subsets. Expression of NKG2D was largely comparable among different CD8^+^ T cell subsets. These observations along with the functional data suggest a differential ability of CD28 and NKG2D in co-stimulating CD8^+^ T cells. These observations for the first time help to establish a functional dichotomy between NKG2D and CD28 receptors.

A reduction of CD28 expression in the CD45RA^+^CD27^−^ effector CD8^+^ T cells lead to the assumption that the effector T cells do not require a co-stimulation and activation via CD3/TCR is sufficient to promote their functions. However, our data indicating an ample expression of MICA in tracheal epithelial cells and the presence of NKG2D on effector CD8^+^ T cells argue that this co-stimulatory system can regulate T cell functions at the site of inflammation. Indeed, our results show that CD28^Lo^ effector CD8^+^ T cells produced higher levels of IFN-γ and TNF-α in response to NKG2D-mediated co-stimulation. Conversely, regardless of its normal levels of expression on CD28^Hi^ CD8^+^ naïve/memory T cells, NKG2D did not augment the production of these inflammatory cytokines in this T cell subset. Our results also indicate that anti-CD28 or anti-NKG2D mAb alone did not result in the production of IFN-γ or TNF-α in the total CD8^+^ T cells or the subsets. Thus, our functional data demonstrate that NKG2D has an expanded co-stimulatory function in the effector CD8^+^ T cells. However, generation of IFN-γ and TNF-α in CD28^Hi^ naïve/memory subset required the co-stimulation via CD28. These observations are supported by earlier studies that have indicated CD28-negative CD8^+^ T cells were more effective in generating IFN-γ during influenza infection [20]. However, this study did not describe the role of NKG2D in these effector CD8^+^ T cell subsets.

NKG2D is a type-II lectin and its cell surface expression requires its association with DAP10 adaptor protein that contains a consensus YXXM tyrosine-based motif [21]. CD28 is a type-I protein and its cytoplasmic tail contains a YINM motif. Activation via NKG2D or CD28 results in the phosphorylation of protein tyrosine kinases (PTK). PTKs phosphorylate YXXM or YINM motifs, which in turn recruits the regulatory subunit of phosphatidylinositol-3-kinase-p85α (PI3K-p85α) that in association with the catalytic subunit PI3K-p110δ transduces downstream signaling [22], [23]. Based on these earlier observations, immediate downstream signaling components appear to be largely shared between NKG2D and CD28 receptors. In this context, it is interesting to note that the generation of IL2 was exclusively dependent on the co-stimulation mediated via CD28. Co-stimulation via NKG2D only minimally induced the generation of IL-2 in effector as well as memory CD8^+^ T cells. This observation highlights the yet unexplored unique molecular signatures of NKG2D and CD28-mediated activation pathways. Cell surface expression of CD107a (Lamp1) that represents the levels cytotoxic granule release did not differ between CD28 and NKG2D-mediated co-stimulation. Hence, we conclude that the CD8^+^ T cell-mediated cytotoxicity is probably not influenced by the type of co-stimulation.

MICA, MICB and ULBPs are stress inducible ligands of NKG2D receptor and they belong to non-classical MHC class I family [1]. Earlier studies have shown the constitutive expression of MIC in intestinal and thymic epithelial cells [24], [25]. Studies have also indicated the constitutive expression of NKG2D ligands in the cytoplasm of human bronchial epithelial cells [26]. However, expression and function of MICs in human tracheal epithelial cells have not been described. Tracheal epithelial cells function as the major barrier to viral infections and thereby become susceptible to influenza virus infections [27]. In the present study, analyses of tracheal and lung specimens obtained from human cadavers indicate that similar to intestinal epithelia, tracheal epithelial cells also constitutively express abundant intracellular MICA. Yet, we were unable to see any significant expression of MICA in cells within the lung tissue. Our study indicates that the MICA was present both on the cell surface and intracellularly in the tracheal columnar epithelial cells. However, a considerable amount of MICA was localized intracellularly. This could be due to the fact that these tissue specimens came from normal healthy individuals. Cellular distress such as viral infections could lead to transport of cytoplasmic MICA to the cell surface. It is also important to note that unlike professional antigen presenting cells, epithelial cells do not express ligands for CD28 [28]. Interestingly, we also observed expression of NKG2D but not CD28 in the tracheal-resident GFL9/HLA-A2 pentamer-positive T cells. Conversely, most of the lung tissue-resident GFL9/HLA-A2 pentamer-positive T cells were both NKG2D and CD28 positive. These findings validate the notion that effector CD8^+^ T cells use NKG2D as their preferred co-stimulatory receptor. Apart from not expressing CD80 or CD86, infected epithelial cells also generate high concentrations of IL15 that is known to influence the expression levels of NKG2D in CD8^+^ T cells [9]. Based on these findings, we conclude recognition of cell surface MICA by CD8^+^ effector T cells via NKG2D could constitute critical co-stimulatory signal that complement the antigenic peptide/MHC-induced TCR activation at the site of infection.

Functions of CD28 as a co-stimulatory molecule have been well established [29], [30]. Naïve and memory CD8^+^ T cells that mostly reside in the lymph nodes (LN) express CD28 along with NKG2D. During influenza infection, tissue dendritic cells (tDC) acquire viral antigens, mature and migrate from the upper respiratory tract to the mediastinal LN, where they activate naïve influenza specific CD8^+^ T cells [31], [32]. Therefore, an increase in the expression of CD80 and CD86 on CDl1c^+^ tDCs is critical to prime via CD28 [33]. The antigen exposure by the dendritic cells converts naïve T cells into effectors, which migrate into trachea to kill virally-infected epithelial cells. At what stage the effector CD8^+^ T cells down regulate CD28 or the molecular mechanism associated with this process is currently not known.

Efficient activation of CD8^+^ T cells is critical to clear tumor or virally-infected cells. Current vaccination strategies are designed to generate and maintain robust memory lymphocyte populations. In contrast, targeted tumor interventions are required to revitalize inefficient effector T cell populations that have failed to clear malignant cells. Thus, these two strategies target two distinct T cell subsets that would require different types of co-stimulatory activations for their immune responses. Based on the present study, we conclude incorporation of co-stimulation via NKG2D along with CD28 is essential and obligatory to stimulate tumor or tissue-infiltrating effector CD8^+^ T cells. However, boosting a recall immune response via memory CD8^+^ T cells or vaccination to stimulate naïve CD8^+^ T cells would require CD28-mediated co-stimulation.

## Materials and Methods

### Cell isolation, sorting and flow cytometry

PBMC were provided from healthy HLA-A2 blood donors by the Sample Core of the Center for Human Immunology and prepared by Ficoll-Hypaque density gradient. Isolation of CD3^+^CD8^+^ T cells was done by negative selection using the CD8^+^ T cell Isolation Kit II (MACS, Miltenyi Biotec). Complete RPMI consisting of 10% FCS, 2 mM L-Glutamine, 100 U/mL penicillin, 100 µg/mL streptomycin and 50 µg/mL 2-mercaptoethanol was used throughout the study. For flow cytometry, cells (2×10^5^) were stained in a final volume of 100 µL of FACS staining buffer (1% bovine serum albumin (BSA), 0.04% w/v sodium azide in PBS, pH 7.2). Antibodies to CD3, CD4, CD8, CD45RA, CD27, CD28 and NKG2D were used at a final concentration of 1∶100. FACS analyses were performed in LSRII (Becton Dickinson) using FACSDiva software. Negative controls were included in the assays wherever required. For sorting CD28^Hi^ and CD28^Lo^ populations, CD8^+^ T cells were negatively selected from PBMC, stained for CD3, CD8 and CD28 and sorted in FACSAria. Human lungs were obtained by the Sample Core from HLA-A2 positive cadaveric organ donors when not needed for transplantion. For isolating lymphocytes, lung tissue was digested with collagenase and DNAse. Lymphocytes from the digested tissue were isolated on a density gradient (Lymphocyte separation medium, MP Biomedicals, Ohio). All protocols used in this study were approved by the BloodCenter of Wisconsin Institutional Review Board (IRB). Informed written consent was obtained in accordance with the Declaration of Helsinki for obtaining human samples.

### T cell activation and quantification of cytokine generation and cytotoxicity

Freshly isolated human PBMC or negatively selected CD8^+^ T cells were stimulated with plate-bound antibodies to CD3 (2.5 µg/mL), CD28 (5.0 µg/mL) and NKG2D (5.0 µg/mL) alone or in combinations. 50 µL of each antibody mix was prepared in ELISA coating buffer (eBioscience, San Jose, CA) and coated on to ELISA plates (Maxisorp, Nunc). 1×10^5^ PBMC or CD8^+^ T cells were added to each well in a final volume of 200 µL. Cells were incubated at 37°C for 18 h and the culture supernatants were collected and cytokines were quantified using 27 or 7 Bio-Plex cytokine assay kits (BioRad, Richmond, CA). For CD107a (LAMP1) surface expression, the T cells were activated in the presence of monensin (1∶1000) and anti-CD107a for 4 h, and stained for the surface expression of CD3, CD8 and CD28.

### Confocal microscopy

Lung and tracheal specimens were treated in 4% paraformaldehyde overnight. Tissue samples (∼1 cm^3^) were embedded in paraffin and 4 µ thick sections were prepared and de-paraffinized in xylene, and re-hydrated. Antigen unmasking was performed by immersing slides in 10 mM sodium citrate buffer, pH 6.0 and heating them at 95°C for 5 min. Slides were allowed to cool in the buffer for approximately 20 min at room temperature. Tissue sections were blocked with 3% BSA and Fc-Block (BD, San Jose, CA) in PBS for 1 h. For analyzing the pentamer-positive cells for their CD28 and NKG2D expression, the sections were incubated with CD8-FITC, NKG2D-APC, CD28-PECy7 and GFL9/HLA-A2 Pentamer-PE at a 1∶100 dilution in 3% BSA overnight at 4°C. For analyzing the expression of MICA, the ligand for NKG2D on epithelial cells, the sections were incubated with pan-cytokeratin (D12, unconjugated mouse monoclonal, Santa Cruz), E-Cadherin (unconjugated rabbit anti-human antibody, Cell Signaling Technology, Beverly, MA) and MICA-APC (R&D systems, Minneapolis, MN) overnight at 4°C. Secondary antibodies to cytokeratin (Goat anti-mouse Alexafluor 568, Molecular Probes, Invitrogen) and E-cadherin (Donkey anti-rabbit Alexafluor 488, Molecular Probes, Invitrogen) were added at a dilution of 1∶500 in 3% BSA. The sections were incubated for 30 min at room temperature and washed with PBS. The sections were then mounted for confocal microscopy (H-1000, VECTASHIELD Mounting medium for fluorescence, Vector Laboratories Inc., San Diego, CA). For analyzing the expression of CD28 and NKG2D on CD4^+^ and CD8^+^ T cells in lung-derived lymphocytes were blocked with 3% BSA and Fc-Block. Cells were then stained with CD8/CD4-FITC, CD28-PECy7 and NKG2D-APC at a dilution of 1∶100 in 3% BSA for 30 min at 4°C. The cells were washed with PBS and suspended in 20 µl of fixing buffer (1% FCS, 2.5% paraformaldehyde in PBS). The cells were mounted on Poly L-Lysine-coated slides. The processed lung and tracheal sections and the lung-derived lymphocytes were visualized using Olympus FluoView FV1000 MPE multiple Laser Scanning Microscope with a 40× oil immersion objective.

### Statistics

Statistical analyses were performed by two-tailed, paired t-test or Student's t-test. *P* values that are ≤0.05 were considered significant.
